# Explaining Food Waste Dissimilarities in the European Union: An Analysis of Economic, Demographic, and Educational Dimensions

**DOI:** 10.3390/foods14244244

**Published:** 2025-12-10

**Authors:** Claudiu George Bocean

**Affiliations:** Department of Management, Marketing and Business Administration, Faculty of Economics and Business Administration, University of Craiova, 13 AI Cuza Street, 200585 Craiova, Romania; claudiu.bocean@edu.ucv.ro

**Keywords:** food waste, economic development, educational attainment, population density, factorial analysis, multilayer perceptron, cluster analysis, European Union

## Abstract

Food waste remains a persistent sustainability challenge for the European Union, revealing how economic development, demographic structures, and educational attainment intersect to shape consumption behavior. Although rising prosperity can enhance efficiency, it often encourages overproduction and habits of abundance that increase food waste. This study investigates the structural drivers behind the variation in per capita food waste across EU member states by examining the combined influences of economic growth, human capital, and population density. Using a cross-country dataset, the analysis integrates factorial methods to identify latent relationships among socioeconomic indicators, a multilayer perceptron to capture nonlinear dependencies, and cluster analysis to classify countries according to shared development and education patterns. The results show that higher income and consumption levels tend to elevate food waste. Nevertheless, this effect is moderated when educational attainment and public awareness are stronger, highlighting the role of knowledge in shaping responsible consumption. The neural network further demonstrates that the relationship between prosperity and waste is not linear but mediated by the cognitive and social capacities of each society. Cluster patterns reveal regional models where sustainability policies and cultural norms contribute to more efficient food management. Overall, the study emphasizes that food waste arises from structural disparities rather than isolated behaviors, offering an evidence-based foundation for integrated EU policies that support more sustainable and equitable resource use.

## 1. Introduction

In recent decades, food waste has grown into one of the most debated issues at the intersection of sustainability, economic development, and social responsibility. Within the European Union, this phenomenon reflects how modern societies negotiate the balance between material prosperity, ecological limits, and ethical considerations. Reports from FAO [1] and UNEP [2] show that nearly one-third of global food production disappears each year, generating substantial economic losses and placing increasing pressure on ecosystems. Researchers increasingly argue that food waste emerges from complex interactions among economic growth, population density, and human capital [3,4,5].

Across the EU, rising incomes and expanding consumer markets reshape lifestyles, supply chains, and expectations regarding food availability. Higher GDP per capita often improves infrastructure and access to resources, yet it also encourages consumption behaviors that lead to overbuying and discarding food [6,7]. Urbanization intensifies this trend, as dense metropolitan areas rely on fast-moving supply chains and convenience-driven shopping patterns that accelerate waste generation [8,9]. These dynamics unfold within a policy environment shaped by the European Green Deal and the Farm to Fork Strategy, which positions food waste reduction as a key component of climate neutrality and circular economy goals.

Although the current literature offers valuable insights into the individual drivers of food waste, most studies still examine these factors in isolation, often emphasizing either economic conditions, demographic pressures, or educational influences without exploring how they interact [10,11,12]. This fragmented approach limits our understanding of the broader structural mechanisms that shape consumption behavior across Europe. Previous research highlights relevant associations, yet it rarely integrates economic development, human capital, and population dynamics into a single analytical framework capable of capturing their combined effect [11,13]. As a result, the field lacks a comprehensive perspective that explains why countries with similar income levels may display very different patterns of food waste, or why educational and demographic characteristics alter the relationship between prosperity and wastefulness [5,10,12]. This gap leaves policymakers without the systemic evidence needed to design interventions that address the underlying socioeconomic and educational roots of the problem.

The present study addresses this gap by examining food waste variations across EU member states through a hybrid methodological approach that brings together factor analysis, a Multilayer Perceptron model, and cluster analysis. By linking economic, demographic, and educational dimensions within the same empirical framework, the research identifies the latent structures that connect these variables and clarifies the mechanisms behind territorial disparities. This integrated perspective provides stronger conceptual grounding for understanding food waste and offers a more reliable basis for evidence-informed policies aligned with the EU’s sustainability goals.

The paper is structured as follows: Section 1 introduces the general context and research motivation; Section 2 reviews key studies on the links among the economy, demography, and food behavior. Section 3 outlines the research methodology, while Section 4 presents the main results. Section 5 interprets the results, and Section 6 summarizes the study.

## 2. Literature Review

Food waste has become a vital topic in academic debates about sustainability and economic performance over recent decades, due to its significant environmental, economic, and social impacts. Within the European Union, this issue is now seen not just as a resource management challenge but as a complex indicator of the connections between economic development, human capital, and demographic makeup. FAO [1] and UNEP [2] state that more than a third of all food produced worldwide—about 1.3 billion tons annually—is wasted. This results in an economic loss of nearly 940 billion USD. Tutar et al. [3] found that systemic economic imbalances are linked to per capita food waste, which directly impacts agricultural performance and macroeconomic stability. Lekavičius et al. [14] indicated that food waste highlights systemic inequalities among EU member states, while Canali et al. [15] and Carvalho et al. [16] emphasized the need for an integrated framework that aligns economic policy, education, and technological innovation. Technological factors, hidden growth drivers, human capital, and demographic distribution all play important roles in influencing food waste levels and territorial convergence within the European Union [17,18]. These factors help explain how economic performance, sustainability, and agricultural productivity are connected.

### 2.1. Food Waste and the Economic, Social and Demographic Factors

Recent studies confirm a strong link between food waste and indicators of economic development such as GDP per capita, consumer spending, and educational attainment. Tutar et al. [3] used the Granger causality test to demonstrate a directional relationship between economic growth and food losses, suggesting that prosperity can lead to less sustainable consumption patterns. Similarly, Gencia and Bălan [4] showed that higher GDP per capita levels are associated with greater household food waste, especially in Western European countries, where overproduction and product diversity contribute to inefficient food management. Wolniak and Grebski [5] also found that these trends worsen in regions with high population density, where food distribution and consumption tend to diverge from actual needs.

Beyond the economic aspect, education significantly influences variations in food waste [19,20]. Other research [21,22,23,24] shows that environmental education and consumer awareness can significantly reduce losses, while higher levels of education are associated with greater food responsibility. Stancu et al. [25] and Porpino [26] support this link, demonstrating that education positively affects shopping planning and waste prevention. According to Ajzen [27], intentions to reduce waste stem from attitudes, social norms, and perceived behavioral control—all of which are closely tied to a person’s education level [28,29,30,31,32]. Slorach et al. [33], Nazli et al. [34], and Wen et al. [35] argue that pro-environmental values and food planning skills are key to sustainable behavior, while Graham-Rowe et al. [36] point out that educational campaigns effectively reduce household waste.

From a demographic perspective, population density increases pressure on food supply chains, influencing waste through factors like urbanization, product availability, and lifestyle trends [6,8,9,37,38]. Simultaneously, the relationship between economic development and food waste is not straightforward, prosperity promotes responsible consumption only when backed by a strong educational foundation [39,40,41].

Empirical evidence consistently shows that food waste negatively affects agricultural performance and natural resource use. Fang et al. [42] and Dogan and Kan [43] demonstrate that food losses increase agriculture’s carbon footprint and weaken the sector’s economic sustainability. Tamasiga et al. [44], Tsimnadis et al. [45], and Tsimnadis and Kyriakopoulos [46] advocate integrating circular economy principles into waste reduction policies. Similarly, Tsimnadis et al. [47,48] emphasize the importance of adopting green technologies and fostering institutional innovation, while Eriksson et al. [49] confirm the direct impact of waste management practices on ecological footprints.

Research consistently shows that reducing food waste relies on the interaction between economic growth, human capital, and demographic changes. These relationships can be effectively measured using factorial models that examine the complex factors influencing food sustainability across the European Union.

Given the complex interdependence among economic development, demographic structure, educational attainment, and consumption behavior, a first hypothesis arises:

**Hypothesis** **H1.** *The level of food waste per capita in European Union countries is significantly associated with factors such as economic development, human capital, and demographic density.*

### 2.2. Convergences and Disparities Among EU Member States

A comparative review of the literature reveals that EU member states can be categorized into distinct groups based on a combination of GDP per capita, population density, consumer spending, and education levels—variables that directly influence average food waste. The studies conducted by Lekavičius et al. [14] and Campoy-Muñoz et al. [50] used models based on Social Accounting Matrices (SAM) to assess the impact of food waste reduction on GDP and employment. Their findings indicate that positive economic effects are more pronounced in countries with strong education systems and well-implemented sustainability policies. The European Commission [51] and the United Nations [52] also demonstrated that integrated macroeconomic techniques can reveal how food waste impacts the economy, jobs, and carbon emissions in indirect ways.

From a territorial perspective, the literature shows that food waste is usually higher in Western Europe. At the same time, Central and Eastern European countries report lower overall levels but suffer greater production losses [7,53]. These differences arise from structural factors—such as infrastructure quality, consumption habits, and logistical effectiveness—as well as cultural and educational differences [54,55,56]. Cluster analyses based on these data confirm the existence of homogeneous groups of countries that combine economic efficiency with social responsibility, with best-practice models from France and Italy often cited as benchmarks [57,58,59].

Recent research [60,61] emphasizes that public policies aimed at reducing food waste can boost economic performance when tailored to national contexts and backed by education and innovation. In this context, the “Farm to Fork” strategy [62] and the food donation directives [63] serve as key pillars of the circular economy, promoting social cohesion and helping to lessen disparities among member states. On the social front, studies by Friman and Hyytiä [64], De Boni et al. [65], and Albizzati et al. [66] show that reducing food waste can create jobs in green and recycling sectors while also enhancing education for sustainability.

From a methodological perspective, cluster analysis serves as a powerful tool for identifying convergence and divergence patterns among EU countries by highlighting unique combinations of economic and educational variables. Xu and Tian [67] argue that this method effectively classifies countries based on structural similarities, providing a solid empirical basis for policy development. Barrera and Hertel [68], Wolniak and Grebski [5], and Calabró and Vieri [69] also confirm that such clusters reflect different levels of economic maturity and the efficiency of food management systems. The relationships among GDP per capita, population density, and consumer spending thus become key to understanding best practices in food waste management [69,70,71]. Furthermore, integrating the principles of degrowth and the circular economy can turn these differences into opportunities for sustainable convergence [72,73,74,75,76].

Building on the hypothesis of territorial patterns in development and food behavior, a second research hypothesis can be proposed:

**Hypothesis** **H2.** *The member states of the European Union form distinct and internally coherent groups when considering their levels of economic development, population density, consumer spending, and educational attainment.*

These groups differ not only in their average per capita food waste but also in the extent to which they display consistent patterns of effective food waste management and examples of best practices.

## 3. Materials and Methods

### 3.1. Research Design

The research design builds on the premise that food waste results from an intricate interplay of economic development, demographic patterns, and educational conditions, rather than from any single determinant. To explore this complexity, the study adopts a comparative framework that examines how these structural factors shape per capita food waste across European Union member states. The objective is to uncover the latent mechanisms behind cross-country variation and to offer an integrated perspective on the similarities and differences that characterize the European context.

The analytical process unfolded through three interconnected stages, each contributing a distinct layer of insight. First, factor analysis served to reveal the underlying structures shared by economic, demographic, and educational indicators, allowing the study to reduce data complexity and identify the common dimensions that shape food waste dynamics. Building on these findings, the second stage relied on a Multilayer Perceptron model to explore the nonlinear relationships among variables. This approach made it possible to capture how development, education level, and population density influence food waste when their effects interact rather than operate independently. The final stage introduced a clustering procedure that classified EU member states into groups with similar socioeconomic profiles. This step provided a broader comparative understanding of how different national contexts combine structural characteristics with distinct patterns of food behavior and resource management.

By integrating these three stages into a single design, the study combines methodological rigor with an interpretive orientation. This approach makes it possible not only to quantify variation but also to illuminate the internal logic that connects development paths, educational profiles, and demographic conditions to the persistence of food waste across the European Union. The dataset was standardized for 2020–2022 to ensure comparability among EU member states and to eliminate variations caused by time differences.

### 3.2. Selected Variables

The empirical analysis draws on a harmonized dataset constructed from official Eurostat sources [77,78,79,80,81], covering all EU member states for the period 2020–2022. The selection of variables reflects the intention to capture, within a single framework, the combined influence of economic development, demographic characteristics, and educational attainment on food waste levels. To ensure transparency and reproducibility, all data were retrieved directly from publicly accessible Eurostat databases, which are referenced both in the bibliography and in the Data Availability Statement.

The dataset construction followed a standardized procedure. After downloading the raw data for each indicator, the values were inspected for completeness and consistency across countries and years. The selected Eurostat series contained no missing values for the 2020–2022 interval, which eliminated the need for imputation and allowed for a fully comparable cross-country analysis. When multiple reporting formats existed for the same indicator, the study used the harmonized Eurostat definition to maintain coherence across member states. All variables were then aligned by year and standardized to ensure compatibility within the statistical analyses.

Food Waste (FW), measured in kilograms per capita, serves as the dependent variable and reflects both resource efficiency and social responsibility in food consumption [3,6]. Population Density (PD), expressed as persons per square kilometer, highlights how spatial concentration shapes consumption patterns and logistical pressures [8]. Gross Domestic Product per capita (GDPpc), measured as a percentage of the EU average, captures differences in purchasing power and development levels. The Percentage of Tertiary Education (PTE), representing ISCED levels 5–8, operates as a proxy for human capital and sustainability awareness [21,34]. Final Consumption Expenditure of Households (FCEH), expressed as a percentage of GDP, provides insight into the share of national income directed toward household consumption.

Table 1 summarizes the variables, datasets, and their sources.

The chosen timeframe captures the most recent fully validated period available across all selected indicators, ensuring a consistent and reliable foundation for the analysis while reflecting an important phase in the EU’s transition toward a more sustainability-oriented economic model.

### 3.3. Methods

The study combines three complementary methods—factor analysis, the MLP model, and cluster analysis—to develop a comprehensive understanding of how food waste relates to the economic, demographic, and educational factors that influence it. The selection of these techniques is driven by the complex nature of the phenomenon, which involves interdependencies that cannot be adequately captured using a single approach.

Factor analysis is the initial methodological step, designed to identify underlying structures among the independent variables. By reducing dimensionality, it groups correlated variables into common factors, uncovering connections that are not visible at the level of individual observations [82]. In this context, factor analysis aimed to identify economic, demographic, and educational factors contributing to variations in food waste across Europe. The method uses correlation coefficients to determine how variables such as GDPpc, consumption expenditure, education level, and population density combine into interpretable factors. Mathematically, the equation can be described as follows:

(1) $$X_{i}=a_{i1}F_{1}+a_{i2}F_{2}+\cdots +a_{im}F_{m}+\epsilon_{i}$$

where

$a_{ij}$—the factor loadings;

$X_{i}$—observed variable (FW, PD, GDPpc, PTE, and FCEH);

$F_{j}$—common factors;

$\epsilon_{i}$—unique errors.

In the second stage, the Multilayer Perceptron (MLP) neural network model estimated and interpreted the complex relationships among the variables identified in the factor analysis. This machine learning method models nonlinear interactions between explanatory factors and the dependent variable, food waste per capita, providing a more flexible analytical framework than traditional statistical models. The MLP’s interconnected layers of artificial neurons identify subtle patterns and hidden interdependencies among economic, educational, and demographic factors, surpassing the limitations of linear models in detecting indirect effects. The general formulation of the network output is expressed as:

(2) $$y_{k}=f(\sum_{j}w_{kj}\;g(\sum_{i}w_{ji}x_{i}+b_{j})+b_{k})$$

where:

$y_{k}$—output variable (FW);

$x_{i}$—input variables (PD, GDPpc, PTE, and FCEH);

$w_{ji}$ and $w_{kj}$—connection weights,

$b_{j}$ and $b_{k}$—biases,

g, f—activation functions.

The strength of this method lies in its ability to capture complex interactions without imposing strict assumptions about data distribution or functional form [83]. Through iterative learning and weight adjustment, the MLP approximates the real behavior of economic and social systems, more accurately reflecting how prosperity, education, and population density interact to shape food waste levels.

In the final stage, cluster analysis grouped EU member states into similar categories based on GDPpc, education level, consumption expenditure, and population density. This method emphasizes both similarities and differences among countries [84], revealing unique patterns of food behavior and resource management. The best approach used was the average linkage method between groups, expressed as:

(3) $$d_{ij}=\frac{1}{kl}\sum_{i=1}^{k}\sum_{j=1}^{l}d(X_{i},Y_{j})$$

where

$X_{1},X_{2},\ldots ,X_{k}$—observations from cluster 1,

$Y_{1},Y_{2},\ldots ,Y_{l}$—observations from cluster 2,

*d*(*X*, *Y*)—distance between subjects,

*k*, *l*—cases (EU countries’ values for FW, PD, GDPpc, PTE, and FCEH).

Together, these methods create a strong methodological framework that combines the analytical power of statistics with the interpretive richness of economic and social research. They enable not only observation but also a deeper understanding of the subtle forces behind the phenomenon, laying a solid groundwork for coherent European policies to reduce food waste.

## 4. Results

### 4.1. Factor Analysis Results and Interpretation

The results of the factor analysis show a clear underlying structure connecting economic and educational factors that affect food waste levels across European Union countries. The KMO coefficient (0.584) indicates a moderate level of sample adequacy, suggesting that the data are sufficiently correlated to extract meaningful factors. Bartlett’s test of sphericity (χ^2^ = 139.720, df = 10, *p* < 0.001) confirms that the correlation matrix differs significantly from the identity matrix, indicating consistent relationships among variables and that factor analysis is suitable for this dataset.

The correlation matrix highlights several key associations (Table 2).

The strongest positive correlation is between GDPpc and the Percentage of Tertiary Education (PTE), with a coefficient of 0.582, indicating a clear link between economic growth and human capital. This finding supports the idea that wealthier economies tend to invest more in education, resulting in a higher share of the population with tertiary degrees. Conversely, the variable for Final Consumption Expenditure of Households (FCEH) shows a strong negative correlation with both GDPpc (−0.823) and education (−0.527). This result suggests that in more developed economies, household spending makes up a smaller portion of GDP, reflecting a more diversified and investment-focused economic structure.

Regarding food waste (FW), the correlations seem weak but are conceptually important. The positive links with GDPpc (0.111) and education (0.205) suggest that waste tends to increase slightly as prosperity and education levels grow—an effect explained by easier access to resources and more food availability. However, these relationships are not strong enough to confirm a direct or simple connection, which is why factor analysis helps reveal deeper underlying links.

The communality analysis confirms that economic variables are the primary contributors to the main factor (Table 3).

DP per capita shows the highest communality (0.674), followed by household consumption expenditure (0.539), while population density and food waste have much lower values (0.259 and 0.068, respectively). This pattern suggests that the extracted latent factor primarily reflects an economic structural dimension, with development level and consumption behavior as the primary drivers of cross-country differences. In the factor matrix, food waste (FW = 0.261) shows a moderate positive loading, indicating that it is somewhat influenced by economic and educational factors, as well as other contextual determinants. The modest loading value suggests that food waste is not directly caused by prosperity but rather an indirect result mediated by consumption habits and social awareness. In more developed economies, high levels of food availability and urban lifestyles may lead to increased waste, although these countries typically have better systems for food recovery and redistribution.

The extracted factor explains approximately 41.7% of the total variance and, despite not accounting for a majority, offers a meaningful foundation for interpreting cross-country contrasts. The polarity of the loadings, positive for GDPpc and tertiary education, and negative for final consumption expenditure, reveals a measurable developmental gradient. At one end of this gradient stand countries with higher GDPpc and more substantial human capital, where educational attainment contributes to more deliberate and efficiency-oriented consumption. At the opposite end lie economies where household consumption represents a larger share of national income, signaling more constrained development paths and a reliance on consumption-driven growth. This contrast provides a more precise, metric-based distinction between structurally advanced economies and those still in earlier stages of economic consolidation.

### 4.2. Testing Hypothesis H1: Nonlinear Relationships Explored Through the MLP Model

The Multilayer Perceptron (MLP) model provides a deeper perspective on how economic, demographic, and educational variables jointly shape food waste patterns across EU member states. The use of an MLP is motivated by the fact that traditional linear regression models assume constant and proportional effects among predictors, which is unlikely in complex socioeconomic systems characterized by interaction effects, nonlinear thresholds, and context-dependent dynamics. Decision trees can capture certain nonlinearities but tend to over-partition small datasets and often fail to model smooth, continuous relationships. The MLP offers a balanced alternative by allowing nonlinear transformations while maintaining a cohesive structure that integrates multiple continuous predictors.

**Figure 1:**
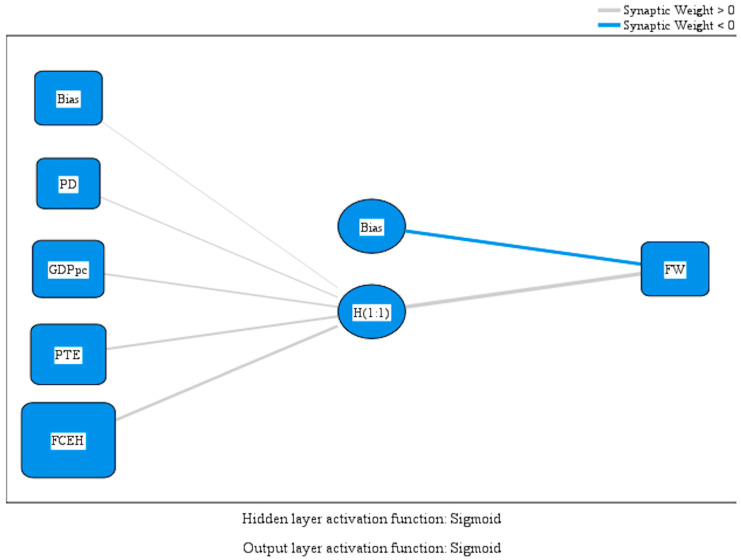
MLP model. Source: author’s design using SPSS v.27 (IBM Corporation, Armonk, NY, USA).

The model includes four input variables: Population Density (PD), Gross Domestic Product per capita (GDPpc), Percentage of Tertiary Education (PTE), and Final Consumption Expenditure of Households (FCEH), feeding into a single hidden neuron. The decision to use one hidden neuron followed iterative model testing aimed at maximizing interpretability and preventing overfitting, given the relatively small sample size of EU-27 observations. Additional neurons increased accuracy only marginally while reducing model transparency and raising the risk of capturing noise instead of meaningful structure. The hidden neuron uses a sigmoid activation function, enabling the network to approximate nonlinear dependencies among predictors, while the output layer uses the same function to generate continuous predictions of food waste (Figure 1).

This configuration results from iterative testing aimed at avoiding overfitting while preserving interpretability. Models with additional neurons increased the risk of memorizing the data rather than learning generalizable patterns, particularly given the limited sample size (EU-27). The single-neuron structure, therefore, serves as a parsimonious compromise, capturing the dominant nonlinear effects without masking them behind excessive model complexity.

The model uses sigmoid activation functions in both the hidden and output layers, allowing the network to approximate smooth nonlinear relationships. The parameter estimates (Table 4) show distinct variations in predictor influence: FCEH exerts the most potent effect on the hidden unit (weight = 0.868), indicating that higher household spending intensifies the behaviors leading to food waste. PTE (0.533) and GDPpc (0.422) also contribute substantially, suggesting that both human capital and prosperity shape consumption dynamics in ways that can either amplify or mitigate waste. PD recorded a smaller yet meaningful influence (0.233), reflecting the more indirect role of urban density and logistical systems.

The normalized importance analysis highlights these key points: Final Consumption Expenditure of Households is the most significant factor (100%), followed by the Percentage of Tertiary Education (64.5%), GDP per capita (57.4%), and Population Density (41.4%). This hierarchy indicates that the structure and level of consumption are the main drivers of food waste, while human capital and economic development shape the relationship between them.

Beyond weight magnitudes, the model reveals important qualitative patterns. For instance, high GDPpc and high education levels do not automatically reduce food waste; instead, they create a social environment in which waste emerges from abundance but can be moderated by learned, sustainability-oriented behaviors. This reveals a threshold effect, in which education gradually offsets the waste-generating tendencies associated with wealth. The MLP’s nonlinear transformation captures this inflection more effectively than linear models, which would obscure the shift from growth-driven waste to awareness-driven efficiency.

The analysis does not imply causality in the strict sense. Instead, it identifies stable associations that align with the results of the factor analysis and contextual interpretations of European food systems. A more detailed sensitivity analysis confirms that adjustments in consumption expenditure and education levels produce the most substantial changes in predicted food waste, while variations in PD and GDPpc generate more moderate effects. This pattern reinforces the idea that the structure of consumption plays a central role, while prosperity and demographic pressures amplify or constrain its impact through interaction effects rather than isolated causal pathways.

Overall, the MLP model validates Hypothesis H1 by demonstrating that food waste results from nonlinear, interdependent relationships. Economic development, human capital, and demographic density do not act separately but shape a multidimensional landscape in which prosperity can encourage wastefulness unless balanced by education, cultural norms, and policy interventions.

### 4.3. Testing Hypothesis H2: Cluster-Based Differences in Food Waste Patterns

Cluster analysis was then used to identify groups of EU member states that are similar based on factors such as economic development, education levels, population density, consumption patterns, and per capita food waste. This exploratory method helps better understand how European countries compare in terms of socioeconomic and behavioral traits that influence their food resource management. Unlike regression or factor analysis, which examine causal links between variables, cluster analysis finds common patterns and relationships, grouping countries with similar features within a clear comparative framework.

The choice of this method is justified due to the complex nature of food waste. The phenomenon cannot be fully explained by economic indicators alone, as it is also shaped by cultural, educational, and demographic factors that differ across regions. Cluster analysis, therefore, serves as a valuable tool for identifying these structural differences, revealing groups of countries with similar development paths and food consumption behaviors.

Table 5 presents the detailed cluster data that support the classification of EU member states and illustrate the structural differences underlying the patterns identified in the analysis.

**Figure 2:**
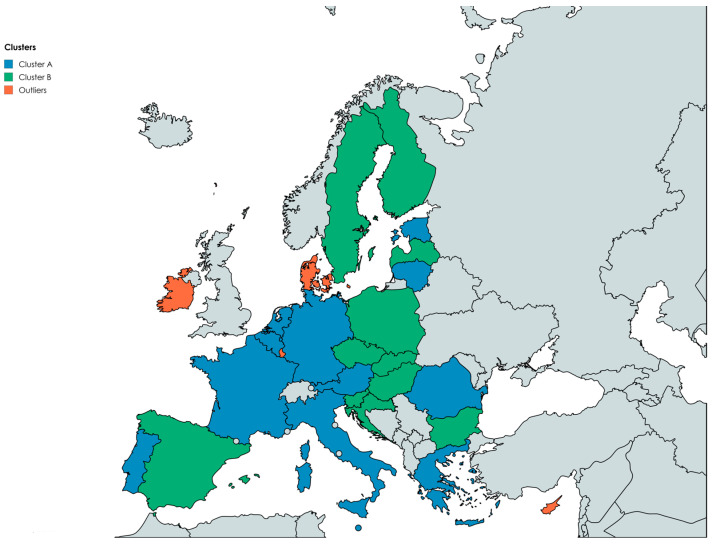
Cluster structure based on FW, PD, GDPpc, PTE, and FCEH. Source: author’s design using mapchart.net (https://www.mapchart.net/europe.html, accessed on 22 October 2025).

By including variables such as GDPpc, the proportion of the population with tertiary education (PTE), final household consumption expenditure (FCEH), population density (PD), and food waste levels (FW), the analysis aims to uncover hidden relationships among economic performance, human capital, and consumption behavior. Figure 2 shows the resulting cluster configuration based on the study’s variables.

The figure shows the grouping of EU countries into two distinct clusters that reflect structural differences in development, consumption patterns, and human capital. Cluster A includes economically advanced or rapidly converging countries, where higher purchasing power and elevated consumption levels tend to generate medium-to-high food waste, although firm educational profiles can moderate these effects. Cluster B comprises countries with more balanced consumption behavior relative to their economic development, indicating that cultural norms, institutional practices, and household spending structures shape food waste dynamics differently.

The division of European states into two main groups, along with a few statistical outliers, highlights the internal consistency of each group and confirms the presence of distinct economic and social trends. Cluster A primarily includes Western and Southern EU nations such as Austria, Germany, France, Italy, Belgium, and the Netherlands, as well as several emerging economies from Central and Eastern Europe, including Romania, Greece, and Portugal. The average metrics for this group (FW = 151.25 kg/capita; GDPpc = 98.38; PTE = 31.36%; FCEH = 55.56%) indicate economies with moderate prosperity and relatively high, though varied, consumption levels. Food waste is generally higher in this group, reflecting a mix of intensive consumption habits and traditional food cultures that are less focused on waste prevention. Southern countries like Greece and Portugal stand out for their high waste levels and above-average spending on consumption, pointing to an economic model still focused on consumption intensity.

In contrast, Cluster B consists of Northern and Central European countries characterized by higher economic and educational efficiency. The average indicators for this cluster (FW = 97 kg per capita; GDPpc = 83.02; PTE = 30.66%; FCEH = 55.80%) show a balanced profile that combines development with social responsibility. Countries like Sweden, Finland, Spain, Slovenia, and the Czech Republic report significantly lower food waste despite having comparable or even higher living standards and food infrastructure. This difference can be linked to stronger educational investment, consistent sustainability policies, and rational consumption habits. In these nations, environmental education and technological innovation in supply chains have been key factors in reducing waste and fostering a culture of resource responsibility.

A key part of the analysis involves identifying outliers—Cyprus, Denmark, Ireland, and Luxembourg—that differ from typical patterns due to unusual combinations of factors. Ireland and Luxembourg, for example, have very high GDPpc (236.6% and 247.9% of the EU average) but do not show corresponding increases in food waste. This result indicates that, beyond wealth, cultural and institutional factors can effectively balance the natural tendency for abundance to lead to waste. Denmark, on the other hand, despite its relatively high food waste levels (254 kg/capita), stands out for its innovative practices in food collection, redistribution, and recycling, exemplifying social and governmental maturity within the circular economy framework.

The results of the cluster analysis show that while GDPpc and consumption expenditure remain important factors in food waste levels, their effects are heavily influenced by educational capital and cultural consumption patterns. Countries in Cluster B demonstrate that a high level of education, combined with sustainability-focused policies, can significantly reduce food loss even in high-income economies. Conversely, countries in Cluster A, where ecological education and prevention infrastructure are less developed, continue to experience higher levels of waste despite economic growth.

In this context, the cluster analysis supports Hypothesis H2 by identifying groups of European countries with similar economic, demographic, and educational characteristics that explain differences in food waste levels. At the same time, the results highlight that statistical similarity does not mean consistent behavior but instead reflects diverse socioeconomic strategies for resource management. Northern and Central European countries emerge as examples of best practices, demonstrating that investment in education, innovation, and civic culture can transform economic growth into a force for sustainability.

In conclusion, confirming Hypothesis H2 emphasizes that reducing food waste in the European Union depends not only on GDP growth but also on how prosperity translates into social and ecological responsibility. The identified clusters reveal that moving toward a sustainable food economy depends on educational maturity and countries’ ability to incorporate sustainability principles into both consumer behavior and public policy.

## 5. Discussions

The results of this research strongly confirm the interdependence among economic development, human capital, and demographic density in explaining variations in food waste across the European Union. The factor analysis identified a clear model where GDPpc and final household consumption expenditure are the primary variables, while education level and population density contribute structural complexity to the phenomenon. These findings support the first hypothesis (H1), which states that per capita food waste levels are significantly affected by factors related to economic development, human capital, and demographic density, while also reinforcing the comprehensive perspective suggested by previous studies [6,14,50].

The relationship between material prosperity and food waste reveals a complex dynamic. Countries with high GDPpc often show elevated waste levels because abundant resources and fast-paced consumption patterns encourage purchasing beyond actual needs. At the same time, these societies tend to invest more consistently in education, which strengthens consumers’ ability to make informed and responsible choices. This interaction between wealth and cognitive capital, highlighted by Borrello et al. [40] and Gencia and Bălan [4], suggests that economic affluence does not automatically generate efficiency. Instead, it becomes effective only when individuals operate within a social environment that values sustainability and provides the knowledge needed to enact it. The MLP model reinforces this idea by showing that income contributes to lower food waste only when education, public awareness, and targeted policy frameworks shape the way prosperity is translated into behavior. This pattern points to an important inference: food abundance alone does not produce waste; waste emerges when abundance coexists with insufficient guidance on how to manage it.

Human capital further deepens this interpretation. Higher levels of tertiary education correlate with more mindful consumption practices and a stronger inclination toward sustainable routines. This finding aligns with the Theory of Planned Behavior [27] and its applications in studies of food-related attitudes [30,31], which show that individuals with greater educational experience tend to form clearer intentions, more substantial normative commitments, and more confident self-regulation in their food choices. Research by Graham-Rowe et al. [36] and Stancu et al. [25] supports this view, noting that education cultivates not only technical knowledge but also a sense of ethical responsibility toward waste reduction. This mechanism suggests that education moderates the influence of prosperity by fostering values that counteract the excesses encouraged by high income levels. As a result, societies with strong educational systems generally manage to decouple economic growth from wasteful behavior.

The cluster analysis offers additional insight into how structural differences shape national profiles of food waste. Cluster A brings together countries such as Austria, Germany, the Netherlands, and France, along with rapidly developing economies like Lithuania and Romania. These cases illustrate contexts where economic abundance and high consumption coexist with medium-to-high food waste. Nonetheless, educational initiatives and growing awareness help reduce the magnitude of waste that would otherwise accompany elevated spending. In contrast, Cluster B, comprising countries such as Spain, Poland, Hungary, and Sweden, shows more balanced behavioral patterns even under moderate economic development. Their combination of reasonable consumption levels, strong civic cultures, and consolidated sustainability norms produces a form of “controlled sufficiency,” which aligns with Xu and Tian’s argument [67] that cultural and institutional typologies shape the outcomes of public programs. These differences imply that reducing food waste depends not only on economic resources but also on how societies organize their values, daily routines, and institutional expectations.

The presence of outliers such as Ireland and Luxembourg provides an especially revealing perspective. Although both countries exhibit exceptionally high GDPpc, their food waste levels do not scale proportionally with their economic standing. This suggests that institutional actions, policy enforcement, and culturally anchored norms can mitigate the pressures typically associated with wealth-induced overconsumption. Vaqué [57] illustrates how legislative frameworks, such as food waste regulations in France and Italy, create behavioral guardrails that prevent high purchasing power from translating into equally high waste. This pattern indicates that policy maturity and civic culture can buffer the environmental consequences of affluence. In these cases, economic growth does not inherently lead to greater waste because institutional environments actively shape the meaning and use of abundance. The evidence therefore supports an important inference: structural conditions, not income alone, determine whether prosperity becomes a driver of waste or a platform for responsible consumption.

The combined use of factor analysis, neural modeling, and cluster analysis offers a comprehensive view of food waste in the EU, consistent with the conclusions of Corrado and Sala [7] and Lekavičius et al. [14], who argue that the issue cannot be addressed in isolation but only within a framework of structural interdependence. Moreover, the findings confirm that economic factors account for only part of the variation in food waste, while educational and cultural aspects play a crucial role in shaping consumption patterns. This insight aligns with the results of Canali et al. [15], who highlighted the importance of behavioral factors in reducing losses throughout the food supply chain.

Compared to previous studies, this research makes a unique contribution by combining quantitative methods with an interpretive analysis of national contexts. Unlike the work of Alexander et al. [17] or Usubiaga et al. [72], which mainly focus on resource flows and ecological externalities, this study offers a socioeconomic and educational perspective on food waste, highlighting the link between prosperity and responsibility. Additionally, the agreement between the MLP results and the cluster analysis suggests a concept of “sustainable maturity”: as European countries develop further, reducing food waste relies not only on material resources but also on shared values and skills.

From a policy perspective, the results support enhancing EU initiatives such as the Farm to Fork strategy [62], emphasizing the need for collaboration between economic and educational frameworks. A sustainable reduction in food waste cannot be achieved through regulation alone but requires fostering a culture of responsible consumption—a process that takes time, cooperation, and innovation. The study thus confirms that the success of public policies depends on their ability to turn knowledge into action and to make education a catalyst for structural change [59]. This perspective reinforces an idea that is increasingly common in contemporary literature [16,60]: reducing food waste is not just an ecological necessity but also a sign of Europe’s social maturity.

### 5.1. Theoretical Implications

The findings from the factor analysis, the Multilayer Perceptron (MLP) model, and the cluster analysis offer a richer theoretical understanding of how economic, demographic, and educational dimensions jointly shape food waste across the European Union. Together, these results support a multidimensional framework in which food waste emerges from the interplay between structural conditions and cognitive capacities, rather than from isolated economic drivers. By demonstrating that education and human capital moderate the influence of economic prosperity on wasteful consumption, the study strengthens a systemic perspective in which sustainability depends on how societies internalize and translate development into responsible behavior.

This interpretation deepens existing connections between sustainable development theory and behavioral models of consumption. It highlights the central role of education as a mediating force that channels economic resources toward more thoughtful and efficient food practices. Consistent with human capital theory [85] and empirical findings on sustainability-oriented behavior [21,30], the results suggest that investments in knowledge reshape not only skills but also values, enabling individuals and communities to align material comfort with ecological responsibility. This dual function of education—as both cognitive and moral capital—broadens the theoretical understanding of how modern societies adjust their consumption habits in response to growing environmental pressures.

Methodologically, the study advances theoretical debates by integrating classical statistical tools with machine learning techniques. The use of the MLP model captures nonlinear relationships that linear approaches typically overlook, illustrating how economic development, educational attainment, and demographic density exert intertwined rather than independent effects. This contributes to the expanding recognition that artificial intelligence can illuminate the complexity of socioeconomic systems, especially those linked to sustainability. The combination of factor analysis, neural modeling, and clustering points to the need for dynamic analytical frameworks capable of reflecting both the structural and behavioral dimensions of food waste. Such an integrated approach enriches theoretical perspectives by showing that sustainable development involves not only economic restructuring but also the cultivation of cognitive and cultural capacities that support long-term resource stewardship.

### 5.2. Practical Implications

From a practical perspective, the research provides vital insights for shaping and implementing European public policies aimed at reducing food waste and promoting sustainability within production and consumption systems. The study highlights connections between GDPpc, education levels, population density, and household consumption expenditure, laying a solid empirical foundation for developing targeted strategies tailored to the specific features of each member state. Grouping EU countries into similar categories allows for a contextualized approach to sustainability initiatives. Countries with high levels of food waste and spending on food could benefit from programs that improve food logistics, run public awareness campaigns, and enhance environmental education. Countries that have successfully integrated economic performance with minimal waste production can serve as models, facilitating knowledge sharing and aligning European sustainability efforts. The results also emphasize how crucial education is as a tool for societal transformation. Policies that prioritize food education, support circular economy projects, and involve schools will help reduce food waste and foster more responsible food habits. This perspective underscores the importance of aligning economic, educational, and cultural policies with the principles of the Farm to Fork Strategy [62] and the European Green Deal.

At the sectoral level, the practical implications also affect the private sector, where the results can help develop business models focused on efficiency and sustainability. Companies in the food industry can use these findings to improve production and distribution processes, reduce losses, and adjust their marketing strategies to align with the changing values of European consumers.

### 5.3. Limitations and Future Research Directions

Although this study provides a comprehensive and well-supported view of the economic, educational, and demographic factors influencing food waste across the European Union, it is important to acknowledge several limitations to ensure a balanced interpretation and to guide future research.

A primary limitation comes from the level of data aggregation. The analysis relies on macroeconomic indicators at the national level, limiting its ability to capture intra-national and regional differences in food-related behaviors. Future research could expand this approach to a microeconomic level by using household or regional data to identify more subtle dynamics and specific drivers in different contexts.

A second limitation relates to the choice of variables. Although the current model is logically sound, it does not include institutional, cultural, or technological factors that could significantly influence food waste. Adding variables related to waste management systems, legislative policies, digitalization, and supply chain innovation would improve future models and provide a more complete understanding of the phenomena.

From a methodological perspective, although the MLP model successfully identified relevant nonlinear interactions, its complexity reduces interpretability. Extending the analysis to include time-series data would allow researchers to examine how food waste evolves and how European policies influence these trends.

Future research could include interregional comparisons between the European Union and other economic groups, such as the OECD or emerging economies, to assess the universality of the proposed explanatory model and identify potential cultural or institutional differences. This approach would strengthen the foundation of a global philosophy of food sustainability and help identify transferable paradigms across regions.

Despite these limitations, this research offers a valuable reference for future studies on the connection between economic development, education, and sustainability. The study’s interdisciplinary approach and combination of quantitative and qualitative methods open new research opportunities and highlight the need for a unified understanding of food waste as a broad social issue at the crossroads of economy, culture, and public ethics.

## 6. Conclusions

This study provides a comprehensive and cohesive analysis of food waste in the European Union, improving both theoretical and empirical understanding of how economic, educational, and demographic factors interact to affect food sustainability. The key idea is that food waste should not be viewed solely as a by-product of economic growth, but rather as a reflection of the alignment of wealth, culture, and social awareness.

The research combines various analytical methods, factorial, neural, and classificatory, to develop an interpretive framework that goes beyond the deterministic view of the direct relationship between income and consumption. The results show that sustainability does not inherently follow from progress, but rather depends on its institutional and cognitive qualities. The model presents that improvements in education and social innovation can transform material abundance into balanced behavior, where consumption becomes a conscious choice rather than a sign of excess.

Epistemologically, the study makes a strong case for reevaluating food waste as a comprehensive social phenomenon that includes economic, ethical, cultural, and psychological aspects. This broad perspective aligns with the increasingly interdisciplinary nature of sustainability research, which seeks to understand not only the material sources of inefficiency but also how values, knowledge, and collective behaviors shape resource use.

In a European context marked by structural changes and pressures on food systems, the study emphasizes the need to shift from reactive to proactive policies—ones based on education, prevention, and cross-sector collaboration. Reducing food waste cannot depend solely on regulation; it requires fostering a culture of responsibility and promoting an economic model that balances efficiency with fairness.

Ultimately, this research promotes a unified view of food sustainability in which economic data, education, and demography are seen as interconnected parts of a single balance system. It emphasizes the transformative role of scientific knowledge in guiding social progress, demonstrating that sustainable development relies not only on economic growth but also on the internalization of shared values such as responsibility, empathy, and solidarity.

The most important contribution of this study is showing that reducing food waste reflects cultural and moral growth more than just seeking efficiency. Its findings indicate that the future relies less on how many resources we have and more on the values we hold, values that will influence how Europe rethinks its priorities between growth and sustainability.

## Figures and Tables

**Table 1:** Variables used, measures, and sources.

Variable	Data	Measures	Sources
FW	Food waste	Kilograms per capita	[77]
PD	Population density	Persons per square kilometer	[78]
GDPpc	Gross domestic product per capita	Percentage of EU27 (from 2020) total per capita (based on million purchasing power standards, EU27 from 2020), current prices	[79]
PTE	Percentage of tertiary education (levels 5–8)	Percentage	[80]
FCEH	Final consumption expenditure of households	Percentage of gross domestic product (GDP)	[81]

Source: author’s design based on Eurostat [77,78,79,80,81].

**Table 2:** Correlation Matrix.

		FW	GDPpc	PTE	FCEH	PD
Correlation	Food Waste (FW), kg/capita	1.000	0.111	0.205	0.043	0.119
	GDP per capita (GDPpc), % of EU average	0.111	1.000	0.582	−0.823	0.126
	Tertiary Education (PTE), % of population	0.205	0.582	1.000	−0.527	−0.039
	Final Consumption Expenditure of Households (FCEH), % of GDP	0.043	−0.823	−0.527	1.000	−0.226
	Population Density (PD), persons/km^2^	0.119	0.126	−0.039	−0.226	1.000
Sig. (1-tailed)	Food Waste (FW), kg/capita		0.163	0.033	0.353	0.144
	GDP per capita (GDPpc), % of EU average	0.163		0.000	0.000	0.131
	Tertiary Education (PTE), % of population	0.033	0.000		0.000	0.366
	Final Consumption Expenditure of Households (FCEH), % of GDP	0.353	0.000	0.000		0.021
	Population Density (PD), persons/km^2^	0.144	0.131	0.366	0.021	

Source: author’s design using SPSS v.27 (IBM Corporation, Armonk, NY, USA).

**Table 3:** Communalities and component matrix.

	Initial	Extraction	Factor 1
Food Waste (FW), kg/capita	1.000	0.068	0.261
GDP per capita (GDPpc), % of EU average		0.674	0.821
Tertiary Education (PTE), % of population	1.000	0.259	0.509
Final Consumption Expenditure of Households (FCEH), % of GDP	1.000	0.539	−0.734
Population Density (PD), persons/km^2^	1.000	0.045	0.211

Source: author’s design using SPSS v.27 (IBM Corporation, Armonk, NY, USA).

**Table 4:** Parameter estimates.

Parameter Estimates					
		Hidden Layer 1	Output Layer	Importance	Normalized Importance
		H(1:1)	Food Waste (FW), kg/Capita		
Input Layer	(Bias)	0.008			
	Population Density (PD), persons/km^2^	0.233		0.157	41.4%
	GDP per capita (GDPpc), % of EU average	0.422		0.218	57.4%
	Tertiary Education (PTE), % of population	0.533		0.245	64.5%
	Final Consumption Expenditure of Households (FCEH), % of GDP	0.868		0.380	100.0%
Hidden Layer 1	(Bias)		−2.057		
	H(1:1)		2.597		

Source: author’s design using SPSS v.27 (IBM Corporation, Armonk, NY, USA).

**Table 5:** Cluster data.

Clusters	Countries	Food Waste (FW), kg/Capita	Population Density (PD), Persons/km^2^	GDP Per Capita (GDPpc), % of EU Average	Tertiary Education (PTE), % of Population	Final Consumption Expenditure of Households (FCEH), % of GDP
Cluster A	Austria	131	109.6	122.9	32.5	50.4
	Germany	129	235.4	120.1	28.0	50.0
	Netherlands	129	517.8	133.8	38.8	43.0
	Belgium	151	383.6	118.2	40.2	48.3
	Estonia	134	31.3	83.7	36.7	51.5
	Lithuania	140	45.2	87.1	41.3	57.0
	France	139	107.6	97.2	36.9	52.2
	Italy	139	198.1	97.6	18.1	59.1
	Malta	162	1696.8	103.1	29.4	49.0
	Portugal	184	115.0	77.0	26.8	68.6
	Romania	181	81.3	73.2	17.1	61.5
	Greece	196	80.3	66.7	30.5	76.1
	Cluster A mean	151.25	300.17	98.38	31.36	55.56
Cluster B	Slovenia	71	104.8	88.7	35.1	55.4
	Spain	65	95.1	87.7	36.5	58.2
	Hungary	84	105.3	76.1	25.6	50.2
	Croatia	72	69.0	71.6	22.8	75.0
	Latvia	124	29.7	69.0	34.5	58.6
	Poland	123	119.8	77.9	30.0	57.3
	Bulgaria	95	58.8	62.1	26.3	59.5
	Slovakia	106	111.5	71.0	26.0	58.3
	Finland	109	18.3	106.4	35.9	48.6
	Sweden	117	25.7	113.7	41.1	44.5
	Czechia	101	138.2	89.0	23.5	48.2
	Cluster B men	97.00	79.65	83.02	30.66	55.80
Outliers	Cyprus	294	100.6	94.3	44.0	62.0
	Denmark	254	140.6	133.6	35.0	43.0
	Ireland	144	75.9	236.6	46.1	23.7
	Luxembourg	122	252.6	247.9	46.0	33.0
	Eu mean	136.89	186.96	103.93	32.77	53.41

Source: author’s design with SPSS v.27 (IBM Corporation, Armonk, NY, USA).

## Data Availability

Research data are publicly available: https://ec.europa.eu/eurostat/databrowser/view/env_wasfw__custom_18351658/default/table?page=time:2022 (accessed on 21 October 2025); https://ec.europa.eu/eurostat/databrowser/view/tps00003/default/table?lang=en (accessed on 21 October 2025); https://ec.europa.eu/eurostat/databrowser/view/nama_10_pc/default/table?lang=en (accessed on 21 October 2025); https://ec.europa.eu/eurostat/databrowser/view/edat_lfse_03__custom_18351776/default/table (accessed on 21 October 2025); https://ec.europa.eu/eurostat/databrowser/view/nama_10_fcs__custom_18351808/default/table (accessed on 21 October 2025).

## Abbreviations

The following abbreviations are used in this manuscript:

MLP	Multilayer Perceptron
FW	Food waste
PD	Population density
GDPpc	Gross domestic product per capita
PTE	Percentage of tertiary education (levels 5–8)
FCEH	Final consumption expenditure of households
